# Socioeconomic and environmental factors associated with glaucoma in an African Ancestry Population: findings from the Primary Open-Angle African American Glaucoma Genetics (POAAGG) study

**DOI:** 10.1038/s41433-024-03470-x

**Published:** 2024-12-11

**Authors:** Anusha Mamidipaka, Amy Shi, Roy Lee, Yan Zhu, Yineng Chen, Isabel Di Rosa, Rebecca Salowe, Gui-Shuang Ying, Joan M. O’Brien

**Affiliations:** 1 Department of Ophthalmology, Scheie Eye Institute, University of Pennsylvania, Philadelphia, PA USA; 2 Penn Medicine Center for Genetics of Complex Disease, University of Pennsylvania, Philadelphia, PA USA; 3 Center for Preventive Ophthalmology and Biostatistics, Perelman School of Medicine at the University of Pennsylvania, Philadelphia, PA USA

**Keywords:** Risk factors, Pathogenesis, Signs and symptoms

## Abstract

**Background/Objectives:**

Glaucoma is the leading cause of irreversible blindness, disproportionately affecting individuals of African ancestry. Limited research has examined the impact of neighbourhood quality and socioeconomic factors on primary open-angle glaucoma (POAG) risk in this population. This study aims to address these gaps by evaluating associations between ocular health and neighbourhood characteristics using geospatial data.

**Subjects/Methods:**

We conducted a case-control study with 5192 African ancestry individuals from the Philadelphia area using data from the Primary Open-Angle African American Glaucoma Genetics (POAAGG) study. Geocoded U.S. Census data were merged with individual-level demographics and neighbourhood-level measures, including air quality, food accessibility, and socioeconomic indicators, to assess their association with glaucoma risk and severity.

**Results:**

The study included 3039 controls (58.5%) and 2153 POAG cases (41.5%). Higher POAG risk was associated with older age (OR 1.72 per 10-year increase, p < 0.001), male gender (OR 2.04, p < 0.001), lower BMI (OR 0.87 per 10 kg/m^2^ increase, p = 0.003), and nonuse of alcohol (OR 0.56 for alcohol use, p < 0.001). Low food access was more common in controls (OR 0.86, p = 0.03), and severe POAG cases were associated with lower homeownership rates (OR 0.95 per 10% increase, p = 0.049). However, most socioeconomic and environmental factors (air quality, education, income, occupation, family structure) were not significantly linked to POAG risk or severity.

**Conclusion:**

Socioeconomic status did not significantly protect against POAG in African ancestry individuals. Individual factors were more influential, suggesting neighbourhood and socioeconomic factors may have a lesser impact than previously hypothesised.

## Introduction

Glaucoma is a group of diseases characterised by optic nerve degeneration and subsequent irreversible vision loss. It is the leading cause of irreversible blindness worldwide, affecting an estimated 2.7 million people in the United States and 70 million worldwide [1]. Primary open-angle glaucoma (POAG), the most common form of the disease, disproportionately affects African ancestry individuals [2]. POAG is five times more prevalent, manifests ten years earlier, and exhibits an accelerated progression to blindness in African Americans compared to European Americans [3]. There has been limited research on the effect of neighbourhood quality and socioeconomic environment on POAG risk in Black individuals.

Neighbourhood quality is defined based on physical and social attributes that can have direct and indirect effects on health outcomes [4]. As a result of enduring systemic and structural racism, neighbourhoods with higher proportions of Black populations often have limited access to quality healthcare facilities, nutritious food options, recreational spaces, and other resources that promote good health [5, 6]. Individuals with chronic health conditions may face increased medical expenses, reduced employment opportunities, and greater financial challenges, which can limit housing options to lower-priced neighbourhoods, leading to higher concentrations of individuals with poor health in disadvantaged neighbourhoods [7]. Areas of low socioeconomic status (SES) are also characterised by elevated risk for adverse all-cause readmission and death after hospital discharge due to exacerbation of chronic diseases stemming from lack of transportation to primary care appointments, inaccessibility of nutritious food, lack of insurance, high medication costs, substance abuse, and depression or other psychosocial barriers to care [8, 9].

Several studies have investigated the association of neighbourhood quality and socioeconomic environment on POAG risk, though Black individuals have been largely excluded from this research. A Taiwanese study revealed that urban living and higher healthcare utilisation increased the likelihood of glaucoma diagnosis, with higher SES individuals more likely to be diagnosed with POAG [10]. A Canadian study, focusing on SES and glaucoma severity, showed that individuals from higher-income neighbourhoods had a marginally reduced risk of presenting with late-stage glaucoma, with the trend particularly noticeable among those aged 65 and older [11]. In a study analysing eye care utilisation among Medicare beneficiaries, glaucoma was found to be more prevalent and severe in Black and Hispanic beneficiaries compared to White beneficiaries [12]. These studies indicate that SES plays a complex role in glaucoma outcomes and healthcare utilisation, with significant disparities observed among racial groups and with variations in how SES affects severity and progression of glaucoma.

To bridge the knowledge gap on environmental effects on POAG risk in Black individuals, our project employs data from the Primary Open-Angle African American Glaucoma Genetics (POAAGG) study at the University of Pennsylvania, which has enroled African ancestry subjects from the greater Philadelphia region. By merging this data with geocoded U.S. Census data at the tract-level, we aim to identify any associations between ocular health and neighbourhood characteristics. Geocoding is the process of assigning spatial coordinates to textual information, which can be used to capture environmental factors [13]. Researchers have utilised census tracts and geocoded data to gain valuable insights into the spatial distribution of health-related factors and to inform targeted interventions to improve community health [14, 15]. Determining risk factors for POAG is particularly important; due to the disease’s relatively asymptomatic early stages, patients with a higher risk of the disease would benefit from regular screening and treatment to slow progression [16]. The goals of this research are to address racial disparities in glaucoma prevalence and severity, and to use geospatial data to identify social and environmental factors that contribute to glaucoma.

## Methods

### Study design and population

The POAAGG study employed a case-control methodology [17]. The study population included self-identified Black individuals (African Americans, African ancestry, or African Caribbean), aged 35 years or older, recruited from the Philadelphia region. Certified Clinical Research Coordinators from the University of Pennsylvania screened potential subjects from comprehensive and subspecialty ophthalmology clinics at the University of Pennsylvania and two neighbouring ophthalmology clinics in Philadelphia (Windell Murphy; Temple University). Excluded from the study were subjects with concurrent ocular disease, including history of narrow angle, closed angle, neovascular, mixed mechanism, or pseudoexfoliation glaucoma; history of glaucoma secondary to eye surgery or secondary to severe ocular trauma; history of iritis, uveitis, or iridocyclitis; presence of Grave’s disease with ocular manifestations, vascular occlusion causing neovascularization of the iris; optic nerve atrophy from other diagnoses; or advanced proliferative diabetic retinopathy. The full inclusion and exclusion criteria are published in the original study [17]. All participating subjects provided informed consent, following the principles of the Declaration of Helsinki, and research was conducted under the oversight of the University of Pennsylvania’s Institutional Review Board.

A glaucoma specialist or ophthalmologist categorised each subject as a case, control, or suspect based on detailed criteria [17]. Cases were identified by the presence of an open iridocorneal angle and characteristic optic nerve defects with corresponding visual field loss, and the exclusion of all secondary causes of glaucoma. For this study, we further divided cases into two groups: “severe” and “non-severe” glaucoma. Severe cases had at least one eye with visual acuity ≤20/200 and/or visual field mean deviation (MD) values ≤ −12 dB [18]. Controls exhibited no significant ocular conditions that could confound the study results.

### Demographic characteristics

At enrolment, Clinical Research Coordinators performed standardised interviews to gather data on systemic illness, behaviour, and demographics. Demographic data included age, gender, self-described race, usage of cigarettes and alcohol, hypertension, and diabetes. Smoking status was categorised into current, former, and never smoked. For analysis, subjects were stratified into two groups: “current smokers” and “non-current smokers.” Alcohol use was classified into two categories: “history of use” and “no history of use.” Electronic medical records were evaluated to determine previous glaucoma surgery history, to supplement onsite examination data, and to obtain current zip code information for each subject.

### Geocoding census data for neighbourhood context

Geocoding is the process of linking health records to census enumeration units, enabling researchers to conduct spatial analyses and to assess relationships between environmental exposures and health outcomes at the neighbourhood level [19]. The boundaries of neighbourhoods are often defined by census blocks and tend to exhibit homogeneity in demographic, social, economic, and environmental conditions [20]. A census tract is a larger geographic area composed of multiple blocks designed to reflect neighbourhoods with similar characteristics [20]. Measures of socio-economic status (SES) and racial composition of the study population were obtained from the 2020 US Census at the census tract level (https://data.census.gov/) [21].

The estimation of subjects’ air pollution exposure based on their county of residence relied on comprehensive data from the Environmental Protection Agency (EPA) [22]. Annual summary files were utilised to access Air Quality Index (AQI) values, calculated daily for PM10, PM2.5, and Criteria Gases. The EPA-established AQI standards for five major air pollutants—ground-level ozone, particulate matter (PM2.5 and PM10), carbon monoxide, sulphur dioxide, and nitrogen dioxide—formed the basis for assessing air quality.

The study employed the Food Access Research Atlas (FARA) from the US Department of Agriculture (USDA) to estimate food access for each subject based on their demographic information and the census tract in which they reside [23]. FARA offers insight into neighbourhood access to healthy and affordable food, enabling users to map census tracts by income status and food store accessibility. By considering store types such as supercentres, supermarkets, and large grocery stores, FARA designates census tracts as low-income and low-access (LILA), analysing both income and access independently. By measuring the share of tract population that are Black or African American that live more than ½ mile, 5 miles, and 10 miles from supermarkets, FARA enables mapping and analysis of low food store access for specific populations, integrating demographic and accessibility factors to understand food disparities and challenges in specific census tracts. Vehicle availability, crucial for assessing food access, was also measured by the USDA. A tract was identified as low access if at least 100 households were more than one-half mile from the nearest supermarket without access to a vehicle; or at least 500 people or 33% of the population lived more than 20 miles from the nearest supermarket, regardless of vehicle access.

### Socioeconomic status estimates

Using data from the 2020 US Census, demographic and socioeconomic information was estimated based on the census tract in which each participant resides. Summary File Three included measures of poverty (percentage of census tract population below the poverty line), unemployment (percentage of census tract population who are unemployed), educational attainment (percentage of persons with at least a high school education), occupation type (percentage of white-collar and percentage of blue-collar employment), disabilities (percentage of census tract population living with disabilities), and health insurance (percentage of census tract population with health insurance) [21]. We also included median family income, housing value, and percentage of owner-occupied units in our study. These measures parallel individual measures of SES included in the model, representing economic stability (median household income) and wealth (percentage of census tract population living in owner-occupied units and median housing value in census tract). Household size (median household size of the census tract), family structure (percentage of married family household and unmarried households in the census tract population), and children (percentage living with children under 17 years old in census tract population) were also measured [21]. These variables offer a broad assessment of the distribution of individuals across housing units within a community (for instance, whether a neighbourhood predominantly consists of individuals living alone), the potential for social interactions within the neighbourhood, and the accessibility of social support [24].

### Statistical analysis

Comparisons between POAG cases and controls and between non-severe and severe POAG groups were analysed using linear regression models for continuous characteristics and logistic regression models for categorical characteristics. Both types of analyses were applied with and without age adjustment. Demographic and socioeconomic estimates were evaluated for all cases and controls within their respective residential census tracts and compared using linear mixed effect models to account for county-level and tract-level correlations. A multivariable regression model was fit by including factors associated with a p < 0.20 in the univariate regression models. The multivariable regression model went through backward variable selection to reach the final multivariable model that only kept statistically significant factors. All statistical analyses were performed with SAS 9.4 (SAS Inc., Cary, NC), and two-sided p < 0.05 was considered statistically significant.

## Results

### Study population

Out of the 5834 eligible POAAGG subjects, 5192 POAAGG subjects (89%) with complete demographic and census information were included in the univariate analysis. Geographically, these subjects came from 1228 block groups and 713 tracts in the greater Philadelphia area. On average, four participants lived in the same block group, and seven lived in the same tract. In terms of disease status, our study cohort comprised 3039 controls (58.5%) and 2153 POAG cases (41.5%). The cases were divided into 1312 non-severe (60.9%) and 841 severe (39.1%) glaucoma subjects.

### Individual-level characteristics, census-tract level demographic factors, and census-tract level socioeconomic factors associated with POAG cases

Supplemental Table 1 compares individual-level demographic characteristics, collected at time of enrolment, between POAG cases and controls. Cases were more likely to: be older (p < 0.001), male (p < 0.001), have a lower BMI (p < 0.001), and have hypertension (p < 0.001). Cases were less likely to: have diabetes (p < 0.001), be a current smoker (p < 0.001), or have a history of alcohol use (p < 0.001). Supplemental Table 2 displays an analysis of census tract-level demographic and SES characteristics, revealing that cases were less likely to: be from areas with high Hispanic or Latino populations (p = 0.001), have children under 17 years old (p = 0.01), or live in tracts with a higher percentage of people living in poverty (p = 0.045). An analysis of neighbourhood characteristics (Supplemental Table 2) revealed that control subjects were more likely to live in census tracts with low food access when accounting for vehicle access (p = 0.009).

In multivariable analysis (Table 1), cases were associated with older age (aOR 1.72 per 10-year increase, 95% CI 1.63–1.82, p < 0.001), lower BMI (aOR 0.87 per 10 kg/m^2^ increase, 95% CI 0.79–0.95, p = 0.003), male gender (aOR 2.04, 95% CI 1.79, 2.33, p < 0.001), absence of diabetes diagnosis (aOR 0.73 for presence of diabetes, 95% CI 0.64–0.83, p < 0.001), nonuse of alcohol (aOR 0.56 for alcohol use, 95% CI 0.49–0.64, p < 0.001), and residence in a census tract without low access to food (aOR 0.86 for every % increase in low food access, 95% CI 0.75–0.99, p = 0.03). Only subjects with complete data for each of these factors associated with POAG were included in the model.

**Table 1:** Multivariable analysis for factors associated with POAG (N = 4981).

Factor	Control [N = 2920 subjects (%)]	Case [N = 2061 subjects (%)]	Adjusted Odds ratio (95% CI)	P-value
Age at Enrolment (per 10-year increase), [mean (SD)]	62.1 (11.7)	70.0 (11.4)	1.72 (1.63,1.82)	**<0.001**
BMI (per 10 kg/m^2^ increase), [mean (SD)]	32.0 (7.5)	29.8 (6.6)	0.87 (0.79,0.95)	**0.003**
Gender, n (%)				
Female	2016 (62.9%)	1191 (37.1%)	ref	
Male	825 (49.5%)	843 (50.5%)	2.04 (1.79, 2.33)	**<0.001**
Diabetes, n (%)				
No	1427 (54.6%)	1188 (45.4%)	ref	
Yes	1414 (62.6%)	846 (37.4%)	0.73 (0.64,0.83)	**<0.001**
Alcohol Use, n (%)				
No	1059 (49.6%)	1077 (50.4%)	ref	
Yes	1782 (65.1%)	957 (34.9%)	0.56 (0.50,0.64)	**<0.001**
Low access to food using vehicle access, n (%)				
No	1855 (56.6%)	1425 (43.4%)	ref	
Yes	986 (61.8%)	609 (38.2%)	0.86 (0.75,0.99)	**0.03**

Table 1. Multivariable analysis of factors associated with primary open-angle glaucoma (POAG) among 4981 subjects (2920 controls and 2061 cases). The p-values presented in bold indicate statistical significance.

### Individual-level characteristics, census-tract level demographic factors, and census-tract level socioeconomic factors associated with POAG severity

Supplemental Table 3 compares individual-level demographic characteristics of non-severe and severe cases. Severe cases were more likely to be older at the age of enrolment (72 vs 69 years, p < 0.001), male (46% vs 38%, p < 0.001), have a lower BMI (29 vs 30 kg/m^2^, p < 0.001), and lack a history of alcohol use (41% vs 51%, p < 0.001). An analysis of census tract-level demographic and socioeconomic characteristics between non-severe and severe cases did not reveal any differences in demographic characteristics but showed that non-severe cases had higher home-ownership rates (52% vs 50%, p = 0.01) (Supplemental Table 4). There were no significant differences in neighbourhood characteristics, specifically air quality and food access, between non-severe and severe cases (Supplemental Table 4).

In a multivariable analysis (Table 2), severe cases were associated with older age (aOR 1.34 per 10-year increase, 95% CI 1.23–1.45, p < 0.001) and current smoking (aOR 1.56, 95% CI 1.19–2.06, p = 0.001) and male gender (aOR 1.67, 95% CI 1.39–2.00, p < 0.001). Non-severe cases were associated history of alcohol use (aOR 0.65, 95% CI 0.54–0.78, p < 0.001), and home ownership (aOR 0.95 per 10% points increase, 95% CI 0.91–0.9997, p = 0.049) (Table 2). Only subjects with complete data for each of these risk factors were included in the model.

**Table 2:** Multivariable analysis for risk factor of glaucoma severity (N = 2077).

Factors	Non-severe Case [N = 1261 subjects (%)]	Severe Case [N = 816 subjects (%)]	Adjusted Odds Ratio (95% CI)	P-value
Age at Enrolment (per 10-year increase), mean (SD)	68.7 (11.4)	72.1 (11.4)	1.34 (1.23,1.45)	**<0.001**
Gender, n(%)				
Female	787 (64.3%)	437 (35.7%)	Ref	
Male	474 (55.6%)	379 (44.4%)	1.67 (1.39, 2.00)	**<0.001**
Alcohol Use, n(%)				
No	617 (56.0%)	484 (44.0%)	Ref	
Yes	644 (66.0%)	332 (34.0%)	0.65 (0.54, 0.78)	**<0.001**
Current Smoker, n(%)				
No	1109 (61.3%)	699 (38.7%)	Ref	
Yes	152 (56.5%)	117 (43.5%)	1.56 (1.19, 2.06)	**0.001**
% owner-occupied units (per 10% points increase), mean (SD)	51.4 (18.3)	49.5 (18.8)	0.95 (0.91, 0.9997)	**0.049**

Table 2. Multivariable analysis of risk factors associated with glaucoma severity among 2077 subjects (1261 non-severe cases and 816 severe cases). The p-values presented in bold indicate statistical significance.

## Discussion

This study examined the relationship between individual factors, SES, neighbourhood characteristics, and POAG severity risk in African ancestry individuals. By highlighting modifiable risk factors, we aimed to devise strategies to mitigate healthcare disparities and prevent early blindness from this familial disease.

Several individual-level risk factors were associated with POAG and/or degree of POAG severity. Lower BMI was associated with increased POAG prevalence, aligning with studies like the Central India Eye and Medical Study and the Rotterdam Eye Study. These studies found that, due to a potential connection with reduced cerebrospinal fluid pressure, lower BMI could be linked to higher POAG risk [25, 26]. Past work in our lab, which stratified BMI into normal or overweight (BMI < 25), overweight (25–<30), obese (> = 30) categories, found that overweight and obese subjects had a lower risk of POAG compared to those of normal range weight [27]. However, this finding is challenging to interpret given the high mean BMI of our cohort and other studies finding a positive BMI-IOP correlation [28]. In our study, control subjects also appeared to have higher diabetes prevalence compared to cases, but this may be due to hospital-based recruitment and exclusion of subjects with proliferative diabetic retinopathy, yielding a subject pool that is less representative of epidemiological diabetes prevalence. Previous research shows mixed findings regarding the POAG-diabetes relationship [29, 30]. The elevated risk and severity of POAG among men likely result from social, biological, and anatomical factors, including distinctions in retinal nerve fibre thickness, ocular structures (axial length, disc area, IOP), and gender disparities observed in other non-communicable diseases like cardiovascular conditions [27, 31]. History of alcohol use was associated with reduced POAG risk and severity. Studies have found that acute alcohol ingestion can result in the short-term reduction of IOP, a recognised risk factor for glaucoma [32, 33]. However, epidemiological studies show inconsistent links between daily alcohol consumption, IOP, and POAG, with both positive, neutral, and negative associations reported [34–36]. Current smoking, compared to never or former smoking, was also associated with more severe POAG, which is supported by meta-analyses highlighting increased POAG risk among smokers [37]. Compromised optic blood flow and vascular issues linked to smoking suggest a relationship between this modifiable risk factor and the vascular pathophysiology that leads to POAG [37]. To note, this study’s categorisation of smoking and alcohol consumption into binary groups of current “use” versus “non-use” may not fully capture an individual’s degree of exposure to these factors. For example, former alcohol users who become abstinent may have underlying health characteristics that differ from lifelong non-users of alcohol [38]. Thus, the interpretation of these findings may be limited and future studies would benefit from more granular classification of substance use history.

Interestingly, control subjects were more likely to live in census tracts with low food accessibility characterised by low vehicle access and sparse grocery stores. Although intake of healthy food is typically associated with fewer health complications, antioxidant intake through consumption of fruits and vegetables did not significantly alter the risk of POAG according to one study [39]. Additional studies of fat intake have found that the total intake of dietary fats did not acutely alter POAG risk, but a high intake in omega-3 fats was associated with a reduced glaucoma risk [40]. Thus, a healthy diet may not seem to affect POAG development in the short-term. However, more research on the relationship between nutrition and glaucoma would be helpful to establish its longitudinal impact on patient outcomes.

After controlling for all other factors, multivariable analysis revealed that subjects with severe POAG were more likely to live in areas with less homeownership [21]. Considering that early stages of glaucoma are asymptomatic, and patients living in areas of low SES tend to have less access to primary care, we might rationalise that subjects who lack the financial stability to own their home may be at higher risk for severe disease [41, 42]. Additionally, renters might be less likely to establish long-term relations with a primary care provider, limiting access to regular eye exams, which are essential for early glaucoma detection [43]. Although our study did not show that household income or education were associated with glaucoma diagnosis or severity, it is still important to consider that patients with low SES are more likely to receive a later diagnosis and present with more severe POAG [44]. Limited financial resources can hinder affordability and timeliness of eye exams, medications, and surgery, contributing to disease progression [45]. Medication adherence is often poor in glaucoma patients, even when treatment is free, particularly among individuals with low SES facing immediate stressors and prioritising basic needs over asymptomatic early-stage glaucoma [16].

While SES plays a significant role in glaucoma disparities among some populations, our study found that SES does not fully explain the observed differences among Black individuals [12]. A 2022 study of Medicare beneficiaries examining eye care utilisation among patients with glaucoma found that differences between Black and White beneficiaries persisted even when frequencies were stratified by SES; in contrast, disparities between other racial groups and White populations were largely explained by SES [12]. The persistence of disparities among Black glaucoma patients, regardless of SES, raises concerns about the role of systemic racism as an independent driver of these disparities. A study on health and income in Black and White populations highlighted “Blacks’ diminished return,” where the protective effects of income against chronic medical conditions were smaller for Black people than for White people [46]. Our study found no significant differences in education, income, occupation-type, or family structure between controls and cases or between non-severe and severe African-ancestry POAG cases. This underscores that high SES might not be protective of glaucoma severity in Black individuals. However, a past study found that low-income, low educational attainment, having glaucoma-related distress, and being unmarried are significantly associated with worse medication adherence among a diverse group of glaucoma patients [47]. While lower SES and lower educational achievement are traditionally linked to a higher glaucoma risk, studies also indicate that individuals with higher income and education tend to be more disease-aware and have better access to healthcare services, potentially leading to a higher representation of these well-resourced glaucoma patients in healthcare systems [48]. These findings call attention to the complicated interplay between SES factors and the need for more epidemiological studies to comprehensively understand how socioeconomic factors impact glaucoma risk, especially in Black populations.

Our study did not find a direct association between glaucoma diagnosis and poor air quality. Glaucoma rates have been found to be 1.5x higher in urban areas with elevated air pollution, which indicates air pollution as a potential risk factor [49]. In other studies, both course particulate matter (PM10, diameter ≤10 microns) and fine particulate matter (PM2.5, diameter ≤2.5 microns) have been associated with increased acute glaucoma risk and more frequent glaucoma outpatient visits [50]. Greater exposure to PM2.5 has been linked to self-reported glaucoma and structural changes in the disease, and particulate matter has been found to promote stress and inflammation in cells [51]. Although this phenomenon may contribute to the overall understanding of how environmental factors can influence eye health, the precise relationship with glaucoma remains complex and multifaceted. Our lack of findings could be due to our limited sample, largely based in an area with similar air quality among most participants.

We recognise this study has several limitations. The cross-sectional nature of the study design limits the ability to establish causal relationships between variables. The reliance on self-reported data for variables such as alcohol consumption and smoking habits introduced the potential for recall bias and may have impacted the accuracy of the results. Additionally, several relevant environmental exposures, such as physical activity and diet, were not included in our analysis. Furthermore, using census tract data to approximate SES and neighbourhood characteristics may not have accurately captured the nuances of specific neighbourhoods, and infrequent Census updates could make it less reflective of current conditions. The use of small-area metrics to assess environmental factors, like air pollution or home ownership status, may not accurately reflect individual exposure levels. Although our study did not find associations with most environmental factors, it is possible that while individual environmental factors have a relatively minor impact on POAG risk, the cumulative effect of multiple factors is more significant. This study may have been underpowered in its population size to detect such small individual effects. Finally, while efforts were made to mitigate selection bias, the study’s recruitment mechanisms, such as through eye clinics and community screening events, might have reached a control population that is more likely to have other (non-POAG) ocular disease or be more motivated or engaged with their health, potentially affecting the representation of the overall population.

In conclusion, the aim of this investigation was to determine the environmental and socioeconomic factors that contribute to the heightened susceptibility of African ancestry individuals to glaucoma, with the overarching goal of reducing healthcare disparities and identifying modifiable factors that extend beyond medication and surgery. Our findings revealed associations between severity of POAG and individual-level risk factors, including BMI, diabetes, alcohol use, and smoking. Notably, the link between severe POAG and lower homeownership underscored the potential impact of socioeconomic factors on disease progression, emphasising the importance of early detection and regular eye exams in vulnerable populations. Despite the common assumption that higher SES provides better access to healthcare resources and should correlate with reduced disease severity, our findings indicate that most socioeconomic and environmental variables alone do not significantly impact the burden or severity of POAG among individuals of African ancestry. Rather, the cumulative effect of multiple risk factors may be more important when comparing individuals living in distinct neighbourhoods. This suggests that the protective effects of higher SES may diminish in relation to POAG, pointing to other factors—such as genetics and heritability, disparities in healthcare access and quality of care, and the effects of chronic stressors linked to systemic and structural racism—that could play a more substantial role in POAG outcomes. To develop targeted interventions, further research is needed to understand and address the social determinants of health that contribute to the significant racial disparity in glaucoma.

## Summary

### What was known before

- Glaucoma disproportionately affects African ancestry individuals, with primary open-angle glaucoma (POAG) being more prevalent and severe in this population compared to European Americans.
- Limited research has explored the impact of neighbourhood quality and socioeconomic factors on POAG risk in Black individuals.
- Previous studies have shown disparities in glaucoma outcomes and healthcare utilisation among racial groups, suggesting systemic racism may contribute to these disparities.

### What this study adds

- Individual-level factors such as older age, male gender, lower BMI, and nonuse of alcohol are associated with higher risk of POAG.
- Most socioeconomic and environmental variables, including air quality, education, income, occupation, and family structure, are not significantly associated with the occurrence or severity of POAG in African-ancestry individuals.

## Supplementary information

Supplemental Tables 1–4 present univariate analyses from the Primary Open-Angle African American Glaucoma Genetics (POAAGG) study.

## Data Availability

The datasets generated during and analysed during the current study are available from the corresponding author on reasonable request.
