# Supplementary material for: Socioeconomic and environmental factors associated with glaucoma in an African Ancestry Population: findings from the Primary Open-Angle African American Glaucoma Genetics (POAAGG) study
Source: Eye (Lond). 2024 Dec 11;39(6):1086–92. doi: 10.1038/s41433-024-03470-x (PMC11978978; doi:10.1038/s41433-024-03470-x)
Supplement: Supplementary file 1 — Supplemental Tables 1–4 present univariate analyses from the Primary Open-Angle African American Glaucoma Genetics (POAAGG) study. [file 41433_2024_3470_MOESM1_ESM.docx]

| **Supplemental Table 1. Univariate analysis of individual-level characteristics of POAAGG subjects (Cases vs Controls).** | | | |
| --- | --- | --- | --- |
| **Variable** | **Control [N=3039 subjects (%)]** | **Case [N=2153 subjects (%)]** | **P-value** |
| Age at Enrollment, [Mean (SD), N] | 62.0 (11.7), 3039 | 70.0 (11.5), 2153 | **<0.001** |
| Age group, n (%) |  |  |  |
| Less than or equal to 60 years old | 1399 (46.0%) | 470 (21.8%) | **<0.001** |
| 60-69 years old | 820 (27.0%) | 546 (25.4%) |  |
| 70-79 years old | 586 (19.3%) | 655 (30.4%) |  |
| Greater or equal to 80 years old | 234 (7.7%) | 482 (22.4%) |  |
| Gender, n (%) |  |  |  |
| Male | 890 (29.3%) | 878 (40.8%) | **<0.001** |
| Female | 2149 (70.7%) | 1275 (59.2%) |  |
| Body Mass Index (BMI), [Mean (SD), N] | 32.0 (7.6), 2993 | 29.8 (6.6), 2123 | **<0.001** |
| Diabetes, n (%) | 1503 (49.6%) | 887 (41.4%) | **<0.001** |
| Hypertension, n (%) | 2206 (72.9%) | 1689 (78.7%) | **<0.001** |
| Current Smoker, n (%) | 499 (16.8%) | 278 (13.2%) | **<0.001** |
| Alcohol Use, n (%) | 1872 (62.6%) | 987 (46.7%) | **<0.001** |
| History of Tobacco Use, n (%) | 1610 (53.9%) | 1162 (54.9%) | 0.48 |

| **Supplemental Table 2. Univariate analysis of Census tract-level Demographic, Socioeconomic, and neighborhood characteristics of POAAGG subjects (Cases vs Controls).** | | | | |  |
| --- | --- | --- | --- | --- | --- |
| **Factors** | **Control (N=3039 subjects), [Mean (SD), N]** | **Case (N=2153 subjects), [Mean (SD), N]** | **P-value*** | |  |
| % black | 69.653 (28.081), 3004 | 70.925 (27.820), 2125 | 0.15 | |  |
| % Hispanic or Latino | 5.672 (7.948), 3004 | 4.967 (6.798), 2125 | **0.001** | |  |
| Median household size | 1.960 (0.413), 3004 | 1.934 (0.428), 2125 | 0.054 | |  |
| % disabilities | 15.154 (5.767), 2907 | 14.946 (5.603), 2079 | 0.23 | |  |
| % children younger than 17 years old | 24.549 (5.681), 2907 | 24.087 (5.649), 2079 | **0.01** | |  |
| % Married | 9.266 (5.200), 3004 | 9.240 (5.025), 2125 | 0.86 | |  |
| Median income | 51798.11 (25987.75), 2997 | 51486.27 (25454.61), 2122 | 0.7 | | |
| Median household value | 167.657 (122.285), 2983 | 168.378 (117.672), 2099 | 0.85 | | |
| % Owner-occupied units | 51.363 (18.200), 3004 | 50.802 (18.485), 2125 | 0.35 | | |
| % Poverty | 20.472 (13.622), 3004 | 19.615 (12.897), 2125 | **0.045** | | |
| Education |  |  |  | | |
| % At least high school graduate | 30.208 (10.348), 3004 | 29.860 (10.215), 2125 | 0.28 | | |
| % At least some college | 27.670 (7.836), 3004 | 27.853 (7.811), 2125 | 0.44 | | |
| % Bachelor’s degree or higher | 26.152 (17.844), 3004 | 26.654 (17.956), 2125 | 0.35 | | |
| % Resident having healthcare insurance | 91.508 (3.643), 3004 | 91.480 (3.704), 2125 | 0.8 | | |
| % White-collar jobs | 44.769 (13.269), 3004 | 44.748 (13.316), 2125 | 0.96 | | |
| Air quality (county-level) |  |  | |  | |
| Median AQI | 44.810 (2.726), 2976 | 44.739 (2.845), 2115 | | 0.42 | |
| Max AQI | 104.289 (5.307), 2976 | 104.310 (5.485), 2115 | | 0.87 | |
| # Days with PM2.5 | 88.940 (40.162), 2976 | 90.217 (41.628), 2115 | | 0.39 | |
| Food access (tract-level) |  |  | |  | |
| Low access tract using vehicle access, [n (%), N] | 1011 (34.6%), 2923 | 638 (30.4%), 2098 | | **0.009** | |
| Share of tract population that are Black or African American beyond 1/2 mile from supermarket | 29.0 (27.2), 1734 | 27.5 (26.9), 1218 | | 0.2 | |
| Share of tract population that are Black or African American beyond 1 mile from supermarket | 7.7 (14.3), 262 | 6.1 (10.9), 196 | | 0.28 | |

| **Supplemental Table 3. Univariate analysis of Individual-level characteristics of POAAGG cases (Mild Cases vs Severe Cases)** | | | |
| --- | --- | --- | --- |
| **Factors** | **Mild Case [N=1312 subjects (%)]** | **Severe Case [N=841 subjects (%)]** | **P-value** |
| Age at Enrollment, [Mean (SD), N] | 68.6 (11.4), 1312 | 72.1 (11.4), 841 | **<0.001** |
| Age group, n (%) |  |  |  |
| Less than or equal to 60 years old | 329 (25.1%) | 141 (16.8%) | **<0.001** |
| 60-69 years old | 352 (26.8%) | 194 (23.1%) |  |
| 70-79 years old | 403 (30.7%) | 252 (30.0%) |  |
| Greater or equal to 80 years old | 228 (17.4%) | 254 (30.2%) |  |
| Gender |  |  | **<0.001** |
| Male | 494 (37.7%) | 384 (45.7%) |  |
| Female | 818 (62.3%) | 457 (54.3%) |  |
| Body Mass Index (BMI), [Mean (SD), N] | 30.2 (6.6), 1294 | 29.0 (6.5), 829 | **<0.001** |
| Diabetes diagnosis, n (%) | 541 (41.4%) | 346 (41.5%) | 0.95 |
| Hypertension Diagnosis n (%) | 1025 (78.3%) | 664 (79.4%) | 0.54 |
| Current smoker, n (%) | 156 (12.2%) | 122 (14.7%) | 0.1 |
| Alcohol Use, n (%) | 651 (50.8%) | 336 (40.5%) | **<0.001** |
| Tobacco Use, n (%) | 692 (53.9%) | 470 (56.6%) | 0.22 |

| **Supplemental Table 4. Univariate analysis of Census tract-level Demographic, Socioeconomic, and Neighborhood characteristics of POAAGG cases (Mild Cases vs Severe Cases)** | | | | |
| --- | --- | --- | --- | --- |
| **Factors** | **Mild Case (N=1312 subjects), [Mean (SD), N]** | **Severe Case (N=841 subjects), [Mean (SD), N]** | **P-value*** | |
| % black | 70.640 (27.916), 1297 | 71.371 (27.679), 828 | 0.6 | |
| % Hispanic or Latino | 4.957 (7.410), 1297 | 4.982 (5.713), 828 | 0.94 | |
| Median household size | 1.938 (0.428), 1297 | 1.928 (0.428), 828 | 0.49 | |
| % Children younger than 17 years old | 24.145 (5.511), 1270 | 23.997 (5.861), 809 | 0.6 | |
| % Disabilities | 14.862 (5.640), 1270 | 15.078 (5.545), 809 | 0.41 | |
| % Married | 9.313 (5.124), 1297 | 9.126 (4.866), 828 | 0.37 | |
| Median income | 52094.05 (25955.40), 1295 | 50534.56 (24635.39), 827 | 0.21 |  |
| Median household value | 167.402 (115.654), 1280 | 169.902 (120.814), 819 | 0.74 |  |
| % owner-occupied units | 51.630 (18.304), 1297 | 49.503 (18.702), 828 | **0.01** |  |
| % Poverty | 19.591 (13.143), 1297 | 19.652 (12.509), 828 | 0.91 |  |
| % Resident having healthcare insurance | 91.398 (3.796), 1297 | 91.609 (3.553), 828 | 0.17 |  |
| Education |  |  |  |  |
| % at least high school graduate | 29.773 (10.290), 1297 | 29.997 (10.102), 828 | 0.61 |  |
| % At least some college | 28.017 (7.780), 1297 | 27.597 (7.856), 828 | 0.23 |  |
| % bachelor’s degree or higher | 26.775 (17.977), 1297 | 26.464 (17.931), 828 | 0.73 |  |
| % White-collar jobs | 44.885 (13.129), 1297 | 44.532 (13.608), 828 | 0.59 |  |
| County-level air quality |  |  |  | |
| Median AQI, | 44.689 (2.904), 1288 | 44.817 (2.750), 827 | 0.19 | |
| Max AQI | 104.194 (5.740), 1288 | 104.491 (5.060), 827 | 0.34 | |
| # Days with PM2.5 | 90.528 (42.023), 1288 | 89.734 (41.025), 827 | 0.36 | |
| Food access |  |  |  | |
| Low access tract using vehicle access and at 20 miles in rural areas, [n (%), N] | 396 (31.0%), 1279 | 242 (29.5%), 819 | 0.49 | |
| Share of tract population that are Black or African American beyond 1/2 mile from supermarket | 28.0 (27.2), 749 | 26.8 (26.5), 469 | 0.47 | |
| Share of tract population that are Black or African American beyond 1 mile from supermarket | 5.7 (10.1), 126 | 6.7 (12.3), 70 | 0.56 | |
